# Aligning goals of for-profit, not-for-profit, and public healthcare organizations by governing for quality: A model for change in the U.S. and other jurisdictions

**DOI:** 10.1177/08404704231160274

**Published:** 2023-03-23

**Authors:** David J. Klein, Jeremy Veillard, Adalsteinn Brown

**Affiliations:** 1 St. Michael’s Hospital, Toronto, Ontario, Canada.; 2 12366 University of Toronto, Toronto, Ontario, Canada.

## Abstract

There has been widespread criticism of privately owned or operated healthcare organizations in Canada and beyond. However, governments have limited resources to infuse the capital and provide the scale necessary to rapidly address the post-pandemic needs of healthcare systems. Ensuring that healthcare providers regardless of ownership or for-profit or not-for-profit status, provide high quality care and ensure health equity is paramount. Here, we propose the use of a governance for quality model based on the Excellent Care for All Act (2010) developed for public hospitals in Ontario for all healthcare organizations regardless of ownership or profit status, to better align all forms of healthcare providers with quality outcomes and equitable and positive patient experience. We believe that this framework is applicable to healthcare organizations both public and private, for-profit and not-for-profit in Canada, the U.S. and beyond.

## Introduction

Prior to the pandemic, healthcare in the United States was a US $3.6 trillion industry amounting to 16.8% of GDP.^1^ The provision of healthcare has evolved over the last decades from the traditional solo practitioner model to a broad array of payors and provider models, with delivery models of increasingly larger scale and complexity governed under private for-profit, private not-for-profit, or public regimes. In this context, for-profit healthcare entities have faced substantial criticism for their perceived pursuit of profit over efforts to improve patient and system level outcomes.

## Discussion

Not surprisingly, the increasing role of private equity investors and large technology companies in the health sector has been a particular lightning rod, although other publicly traded or private-for-profit entities have not been immune from this critique. Recent headlines have seen Amazon acquire One Medical for US $3.8 billion and the US $17 billion buyout of healthtech company Athenahealth by Bain Capital and Hellman & Friedman in 2021.^2,3^ Although some of these deals have shown success in the past both for investors and arguably for patients, others have been heavily criticized or even have completely failed including the high-profile examples of Haven Health backed by Amazon, JP Morgan and Walmart, or the Hahnemann hospital debacle in Philadelphia recently chronicled in *The New Yorker*.^4,5^ Several strategies have been proposed to help address these concerns including shifting to value-based care payment models to improve alignment between financial, patient, and system outcomes.^6^ While implementing value-based payment models will take time to scale and will require a massive re-engineering on the part of both provider and payor communities, more should and can be done immediately through the application of simple yet effective governance and quality systems. We argue that these mechanisms can be implemented relatively simply across all types of providers and ownership models without creating excessive red tape or government oversight.

One relatively cheap, concrete, and evaluated intervention comes from legislation in Ontario, Canada, that increases market information while focusing executives on quality. The concepts contained in legislation are not new in the US with organizations like the Cleveland Clinic having publicly reported on outcomes since the 1950s, or and tools like the Quality Assurance and Performance Plans (QAPI) implemented in some nursing homes and for specific provider agencies by the CMS.^7^ However, they are not universally required and rarely rise to the level of public, regulator, or investor scrutiny that drives organizational change. These changes, surprisingly, are less burdensome than what we already expect from publicly traded companies that do not deal directly in healthcare.

In Ontario, Canada, a large single payer public healthcare market covering over 15 million lives, we and others sought to improve the alignment between health quality and outcomes with financial performance and compensation. This took the form of a legislative bill entitled the *Excellent Care for All Act* (ECFA) passed into law in Ontario in 2010 with unanimous support of all political parties and legislators.^8^ The act sought to achieve such alignment by requiring all healthcare organizations to: (i) develop and post annual quality improvement plans, (ii) implement patient and employee satisfaction surveys and a patient relations process, (iii) link executive compensation to the achievement of targets in the institutional quality improvement plans, (iv) create quality committees to report to each hospital board on quality related issues, and (v) develop declarations of values after public consultation for each organization.

Qualitative evaluations of ECFA since its implementation have shown that it has led to an intense focus on quality improvement activities, particularly in institutions that had not prioritized quality of care prior. The Act also led organizations to improve their governance mechanisms including better representation of quality expertise on boards of directors to better scrutinize and contribute to quality related outcomes and set stretch metrics for quality outcomes. One evaluation by Kutty et al. noted that in the first year after ECFA, the development of quality improvement plans (QIP) led to significant changes in the prioritization of improvement by over 60% of healthcare institutions. The authors identified efforts by healthcare organizations to standardize QIPs based on the US Institute for Healthcare Improvement model to (i) support hospitals in being compliant with the legislation and related regulations; (ii) be easy to interpret, and provide a snapshot view of quality; (iii) be generalizable to all hospitals, regardless of size or type; (iv) create a Quality Improvement Plan (QIP) that is standardized and comparable across the province, with a core set of indicators that are relevant to all hospitals; (v) create a QIP that is unique enough to each hospital to allow room for indicators that are especially relevant to a particular region or centre; and (vi) streamline the reporting requirements of hospitals.^9,10^

Our proposal is straightforward. We propose creating an ECFA-like governance and quality framework for ALL healthcare organizations public or private, large, or small in the US or other similar jurisdictions. Organizations would be required to develop annual QIPs, have board level quality committees, publicly report on performance against regional and/or national standards and evaluate and compensate management and boards based on quality performance metrics, in addition to financial metrics. Although this type of activity may be seen by some as an undue regulatory and reporting burden, we argue that embracing this approach could improve organizational efficiency by focussing activities on areas of most imminent need that are most linked to patient experience and outcomes. This will further contribute to institutional and system financial sustainability over time. We further believe that this step is a necessary and important waypoint as healthcare organizations move into value-based organizational models. Such a proposal is easy to implement and provides much needed transparency that is valuable to regulators, patients, and investors without inserting the government as a manager. Implementation may not even require legislative change and could be led by agencies such as the Institute for Healthcare Improvement (IHI), expansion of the Centre for Medicare & Medicaid Services (CMS) “Compare” program or the Agency for Healthcare Research and Quality (AHRQ) or even be led by large corporations or industry associations themselves as a potential competitive differentiator. Furthermore, it is consistent with private sector core strengths such as measurement and reporting of a range of indicators including financial indicators, which are critical for the long-term financial sustainability of investors in healthcare in the US. In essence, this approach should strengthen markets for healthcare by increasing transparency and a focus on performance that address broader goals that might easily be missed with a short-term focus on returns only.

These requirements can also help improve organizational and system outcomes by ensuring clear goals for improvement and both executive (because of the payment requirements) and public attention to these goals. In evaluations of the ECFA, this relationship appears to bear out. However, challenges like needing to make sure that the data is clear and easy to access, understanding how to make comparisons to trigger some of the impact of public reporting, as well as the reputational risk of poor performance will need careful consideration for local, regional, and national officials.

## Conclusion

Although all healthcare organizations continue to face challenges around cost, quality, and innovation, we believe that the proposed model can help set a level playing field for all healthcare organizations regardless of size or profit motive working towards a common aim to improve patient and system outcomes for all.
